# Supplementary material for: Impact of exposure to tobacco smoke, arsenic, and phthalates on locally advanced cervical cancer treatment—preliminary results
Source: PeerJ. 2016 Sep 8;4:e2448. doi: 10.7717/peerj.2448 (PMC5018676; doi:10.7717/peerj.2448)
Supplement: Table S1 — LOD, method limit of detection; MBP, mono butyl phthalate; MBzP, mono benzyl phthalate; MEHP, mono (2-ethylhexyl) phthalate; MEOHP, mono (2-ethyl-5-oxohexyl) phthalate; MEHHP, mono (2-ethyl-5-hydroxyhexyl) phthalate; %MEHP = 100 × (MEHP/(MEHP + MEOHP + MEHHP)) on a molar basis [file peerj-04-2448-s001.docx]

| **Analyte** | **Min.** | **25^th^ %tile** | **50^th^ %tile** | **75^th^ %tile** | **Max.** | **Geometric mean** |
| --- | --- | --- | --- | --- | --- | --- |
| Cotinine | < LOD | < LOD | 15.7 | 53.1 | 281.9 | 20 |
| Arsenic | < LOD | 12.4 | 18.1 | 32.6 | 202.5 | 18.9 |
| MBP | < LOD | < LOD | 15 | 45.6 | 1098 | 12.6 |
| MBzP | < LOD | 2.5 | 6.4 | 16.7 | 138.9 | 7.1 |
| MEHP | 2.5 | 12.3 | 21.4 | 40.1 | 265.8 | 22.4 |
| MEOHP | < LOD | < LOD | 3.8 | 14.3 | 48.9 | 5 |
| MEHHP | < LOD | 5 | 15.2 | 24.7 | 279 | 12.4 |
