# Supplementary material for: Impact of exposure to tobacco smoke, arsenic, and phthalates on locally advanced cervical cancer treatment—preliminary results
Source: PeerJ. 2016 Sep 8;4:e2448. doi: 10.7717/peerj.2448 (PMC5018676; doi:10.7717/peerj.2448)
Supplement: Table S2 — a Adjusted for baseline tumor size (cm), age (years), and urinary cotinine for arsenic and phthalates (μg/g creatinine); b %MEHP = 100 × (MEHP/(MEHP + MEOHP + MEHHP)) on a molar basis. [file peerj-04-2448-s002.docx]

| **Predictors** | **n** | **β** | **95% CI** | | **P-value** |
| --- | --- | --- | --- | --- | --- |
| Cotinine | 37 | -0.002 | -0.006 | 0.001 | 0.161 |
| Arsenic | 37 | 0.005 | -0.002 | 0.013 | 0.151 |
| MBP | 35 | 0.0006 | -0.001 | 0.002 | 0.458 |
| MBzP | 35 | -0.002 | -0.014 | 0.009 | 0.686 |
| MEHP | 35 | 0.002 | -0.003 | 0.007 | 0.489 |
| MEOHP | 35 | -0.008 | -0.032 | 0.017 | 0.525 |
| MEHHP | 35 | 0.001 | -0.005 | 0.007 | 0.664 |
| **%MEHP ^b^** | **35** | **0.014** | **0.002** | **0.026** | **0.021** |
